# ICTV Virus Taxonomy Profile: *Arteriviridae* 2021

**DOI:** 10.1099/jgv.0.001632

**Published:** 2021-08-06

**Authors:** Margo A. Brinton, Anastasia A. Gulyaeva, Udeni B. R. Balasuriya, Magda Dunowska, Kay S. Faaberg, Tony Goldberg, Frederick C. C. Leung, Hans J. Nauwynck, Eric J. Snijder, Tomasz Stadejek, Alexander E. Gorbalenya

**Affiliations:** ^1^​ Georgia State University, Atlanta, USA; ^2^​ Leiden University Medical Center, Leiden, the Netherlands; ^3^​ School of Veterinary Medicine, Louisiana State University, Baton Rouge, LA, USA; ^4^​ Massey University, Institute of Veterinary Animal and Biomedical Sciences, Palmerton North, New Zealand; ^5^​ Agricultural Research Service, USDA, Ames, IA, USA; ^6^​ School of Veterinary Medicine, Madison, WI, 53706, USA; ^7^​ The University of Hong Kong, Hong Kong SAR, PR China; ^8^​ The Jockey Club College of Veterinary Medicine and Life Sciences, City University of Hong Kong, Hong Kong SAR, PR China; ^9^​ Ghent University, Gent, Belgium; ^10^​ Institute of Veterinary Medicine, Warsaw University of Life Sciences, SGGW, Warsaw, Poland; ^11^​ Lomonosov Moscow State University, Moscow, Russia

**Keywords:** *Arteriviridae*, ICTV Report, taxonomy

## Abstract

The family *Arteriviridae* comprises enveloped RNA viruses with a linear, positive-sense genome of approximately 12.7 to 15.7 kb. The spherical, pleomorphic virions have a median diameter of 50–74 nm and include eight to eleven viral proteins. Arteriviruses infect non-human mammals in a vector-independent manner. Infections are often persistent and can either be asymptomatic or produce overt disease. Some arteriviruses are important veterinary pathogens while others infect particular species of wild rodents or African non-human primates. This is a summary of the International Committee on Taxonomy of Viruses (ICTV) Report on the family *Arteriviridae*, which is available at ictv.global/report/arteriviridae.

## Virion

**Fig. 1.**
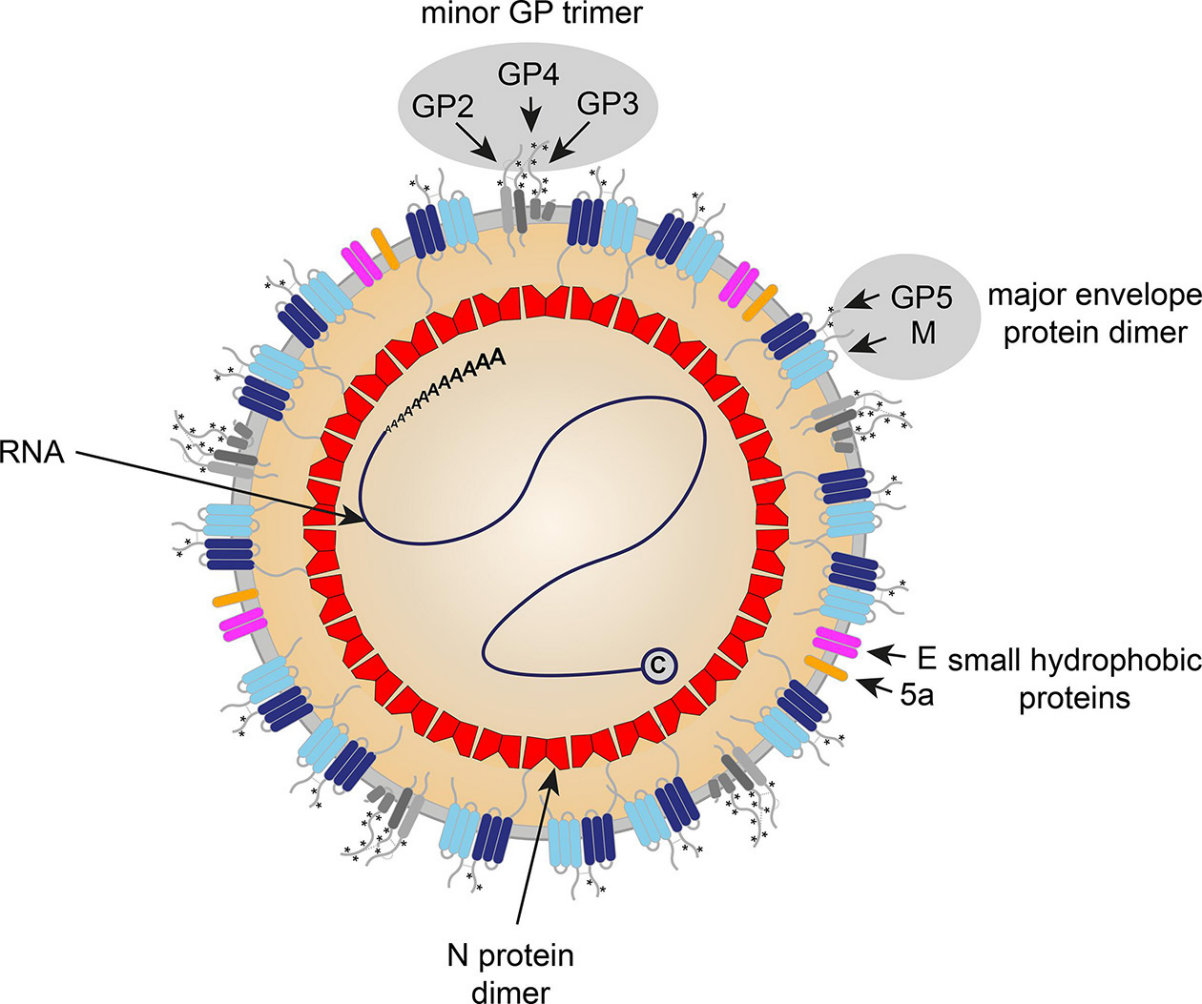
Arterivirus virion structure. C, cap.

Virions are pleomorphic but roughly spherical (diameter 50 to 74 nm). Nucleocapsid protein dimers form a roughly spherical capsid of about 30 nm in diameter lacking icosahedral symmetry [1]. The capsid is surrounded by a lipid envelope derived from the infected cell. Small surface projections, consisting of the major viral envelope protein glycoprotein 5 and the associated membrane protein, cover the virion surface. The virion surface also contains complexes of the minor structural proteins (short spikes) (Table 1, Fig. 1).

**Table 1.** Characteristics of members of the family *Arteriviridae*

Example:	equine arteritis virus (X53459), species *Alphaarterivirus equid*, genus *Alphaarterivirus*
**Virion**	Pleomorphic but roughly spherical particles of 50 to 74 nm in diameter
**Genome**	Linear, positive-sense RNA of 12.7 to 15.7 kb
**Replication**	The viral RNA is replicated in cytoplasmic double membrane vesicles by the ribonucleoprotein transmembrane complex
**Translation**	Cytoplasmic, from viral capped and poly-adenylated genomic and subgenomic mRNAs
**Host range**	Vertebrates, predominantly non-human mammals
**Taxonomy**	Realm *Riboviria*, kingdom *Orthornavirae*, phylum *Pisuviricota*, class *Pisoniviricetes*, order *Nidovirales*, suborder *Arnidovirineae*; the family includes >5 subfamilies, >12 genera, >10 subgenera and >20 species.

## Genome

**Fig. 2.**
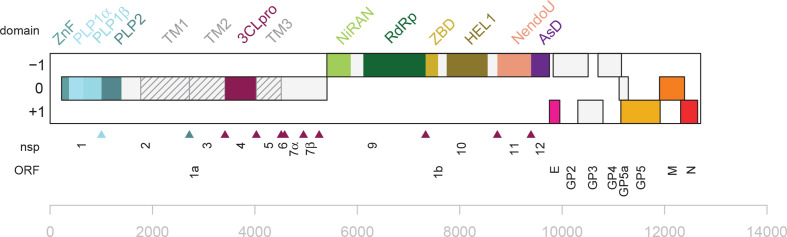
Genome organization and replicase protein domains of equine arteritis virus. The pp1a-encoded nsp8 is not labeled. ZnF, Zn-finger; PLP1α, PLP1β, PLP1γ, and PLP2 are various papain-like proteases; TM1, TM2 and TM3, three transmembrane domains; TM1 includes a Cys/His-rich C-terminal domain; 3CLpro, 3C-like protease; NiRAN, nidovirus RdRp-associated nucleotidyltransferase; RdRp, RNA-directed RNA polymerase; ZBD, Zn-binding domain; HEL1, superfamily one helicase; NendoU, nidovirus uridylate-specific endonuclease; AsD, arterivirus-specific domain.

The genome is a single molecule of positive-sense RNA ranging from 12.7 to 15.7 kb (Fig. 2) with a 5′-type I cap and a 3′-terminal poly(A) tract that is infectious when transfected into permissive cells. Most arterivirus genomes have 10–11, mostly overlapping, functional ORFs, but viruses in the subfamily *Simarterivirinae* have genomes with 15 ORFs, due to a tandem duplication in the minor structural gene region.

## Replication

After virion attachment, endocytosis and membrane fusion, the genome is released into the cytoplasm. The largest (overlapping) ORFs, 1a and 1b are translated to produce the pp1a polyprotein, and after frameshifting, pp1ab. Polyproteins are autoproteolytically cleaved by several viral PL proteases and a 3 CL protease into 13 to 17 mature nonstructural proteins [2] that induce double-membrane vesicles and form the associated viral RNA replication/transcription complexes. These include the NiRAN, RdRp and HEL1 enzymes, which catalyse viral RNA synthesis. Full-length negative-sense RNA is the template for genome replication. Subgenomic negative-sense RNAs are produced by a discontinuous transcription mechanism and function as the templates for subgenomic mRNAs encoding the structural proteins. The various subgenomic mRNAs contain different lengths of 3′-sequence as well as the 5′-leader sequence. Nucleocapsid dimers and a nascent genome RNA associate to form roughly spherical capsids in the cytoplasm, which then bud into the lumen of the endoplasmic reticular membrane at regions with inserted viral envelope proteins. Virions are transported through the secretory pathway and released by endocytosis. Arterivirus infections in non-human mammals are often persistent and can be asymptomatic or produce overt disease.

## Taxonomy

Current taxonomy: www.ictv.global/taxonomy. Since the ninth report, arterivirus taxonomy has advanced several times [3, 4]. The current classification is based on DEmARC analysis of the 3CLpro, NiRAN, RdRp, ZBD and HEL1 protein sequences that are also conserved in other nidoviruses [5]. Local minima in the clustering cost distribution of pairwise distances were used to delimit four ranks and demarcate monophyletic taxa. The family *Arteriviridae* belongs to the suborder *Arnidovirineae* of the order *Nidovirales* that also includes the families *Coronaviridae, Tobaniviridae, Mesoniviridae* and *Roniviridae* and nine others. Nidoviruses share a similar genome functional organization and expression strategy, and each family forms a monophyletic cluster. Compared to members of other families in the order, arterivirus genomes are the smallest, and are unique in virion size, structure and composition.

## Resources

Full ICTV Report on the family *Arteriviridae*: www.ictv.global/report/arteriviridae.

## References

[1] Snijder EJ, Kikkert M, Fang Y (2013). Arterivirus molecular biology and pathogenesis. J Gen Virol.

[2] Li Y, Tas A, Sun Z, Snijder EJ, Fang Y (2015). Proteolytic processing of the porcine reproductive and respiratory syndrome virus replicase. Virus Res.

[3] Faaberg KS, Balasuriya UB, Brinton MA, Gorbalenya AE, Leung F-C, King A, Adams M, Carstens E, Lefkowitz E (2012). Virus Taxonomy, the 9th Report of the ICTV.

[4] Kuhn JH, Lauck M, Bailey AL, Shchetinin AM, Vishnevskaya TV (2016). Reorganization and expansion of the nidoviral family *Arteriviridae*. Arch Virol.

[5] Gulyaeva A, Dunowska M, Hoogendoorn E, Giles J, Samborskiy D (2017). Domain organization and evolution of the highly divergent 5’ coding region of genomes of arteriviruses, including the novel possum nidovirus. J Virol.

